# Effects of Fermentation Temperature and Time on the Color Attributes and Tea Pigments of Yunnan Congou Black Tea

**DOI:** 10.3390/foods11131845

**Published:** 2022-06-23

**Authors:** Jiayi Zhu, Jinjin Wang, Haibo Yuan, Wen Ouyang, Jia Li, Jinjie Hua, Yongwen Jiang

**Affiliations:** 1 Tea Research Institute, Chinese Academy of Agricultural Sciences, Hangzhou 310008, China; zhujiayi@tricaas.com (J.Z.); jinjinwangtkzc@tricaas.com (J.W.); 192168092@tricaas.com (H.Y.); owy980501@193.com (W.O.); jiali1986@tricaas.com (J.L.); 2 Key Laboratory of Tea Quality and Safety Control, Ministry of Agriculture and Rural Affairs, Hangzhou 310008, China

**Keywords:** Yunnan Congou black tea, fermentation, temperature, tea pigment, color attribute

## Abstract

Yunnan Congou black tea (YCBT) is a typical black tea in China, and is rich in theaflavins (TFs), thearubigins (TRs), and theabrownins (TBs). However, the influence of the fermentation temperature and time on the liquor and appearance color and the correlation between the tea pigments and its color attributes remain unclear. We investigated the effects of the fermentation temperature and time on the color attributes and tea pigments of YCBT. A low fermentation temperature was beneficial to maintain a bright orange-red liquor color and promote the accumulation of TFs and TRs. In contrast, a high temperature gave the liquor a glossy appearance and was beneficial for the formation of TBs. A correlation analysis showed that the 10TFRB index best represented the contribution of tea pigments to the quality of black tea. Moreover, TRs and TBs content prediction models were established based on the liquor *L* and *H* values, where the former value can be used as an important index to judge the fermentation process. This study will further enrich the theory of black tea processing chemistry and provide technical support for the precise and directional production of black tea.

## 1. Introduction

Tea, one of the most popular drinks worldwide, is processed from the young, tender shoots of *Camellia sinensis* (L.) *O. Kuntze*. Black tea is the most widely produced and consumed tea product due to its unique flavor, taste, and color [1], which are important indicators when judging the quality of black tea and its corresponding market price [2]. Based on the properties of fresh tea leaves, black tea in China can be divided into medium/small-leaf types, such as Qimen black tea, Yihong, and Chuanhong; and large-leaf types, such as Dianhong, Yinghong, and Guihong [3]. Different tea varieties contain different biochemical components in their fresh leaves [4], and these components can greatly influence the sensory quality of black tea [5]. For example, the large-leaf species Yunnan Congou black tea (YCBT) has a mellow taste [6] and a fresh and strong aroma [7], while the lobular species Qihong has a fresh taste and a sweet fragrance [8]. The production area of black tea in China is vast, and the style characteristics of the black teas produced in different geographical locations are known to differ significantly. Since Yunnan is one of the most important tea regions globally [9], and the large-leaf tea trees are rich in germplasm resources [10], it is of particular interest to explore the rational development, utilization, and processing technologies of YCBT to provide a theoretical basis for the production of high-quality black tea.

The processing of black tea generally includes withering, rolling, fermentation, first drying, and second drying, wherein fermentation plays a crucial role in determining the black tea quality. Different from traditional beer fermentation, the tea fermentation is a series of reactions of components in leaves under the action of enzyme activity at a certain temperature and humidity [11]. Under the action of polyphenol oxidase and peroxidase, catechins are oxidized to form quinones, which are subsequently oxidized and condensed to form theaflavins (TFs), theasinensins (TSs), thearubigins (TRs), and theabrownins (TBs) [12,13], which greatly influence the taste, liquor, appearance, infused color, and biological properties of the final tea samples [14,15]. Temperature is known to be one of the key parameters of the fermentation process, and it has been reported to directly affect the conversion of catechins and the formation of TFs, TRs, and TBs [16]. At present, the majority of studies related to black tea fermentation have focused on the variation of external factors, such as the temperature [17,18], humidity [19], time [20], and oxygen supply [21,22], to control the degree and quality of fermentation. In addition, studies on the fermentation temperature have mainly investigated its influence on the taste substances [23] and aroma components [18], and to the best of our knowledge, the effects of temperature on the change of liquor and appearance color and the formation of tea pigments during the fermentation of YCBT have not yet been studied in detail. Moreover, the evaluation of the moderate fermentation process continues to be based on the subjective judgment of tea makers [16]. Therefore, it is necessary to clarify the factors responsible for determining the quantitative indexes related to the liquor color attributes during YCBT fermentation, screen the key color indexes, and establish a prediction model for quality components based on the objective color attribute indexes. The development of such quantitative guidelines would be indispensable in terms of providing support for the accurate processing of black tea fermentation.

Thus, we herein report our investigation into the effects of different fermentation temperatures (i.e., 25, 30, and 35 °C, and natural fermentation (23–30 °C, control check, CK)) and fermentation times (2.5, 3.5, 4.5, and 5.5 h) to explore the influences of these parameters on the key quality components (i.e., the TFs, TRs, and TBs), and the liquor color/appearance attributes (i.e., *L*, *a*, *b, C*, and *H*) of YCBT. For this purpose, we employed ultraviolet (UV) spectrophotometry and a portable optical color meter to detect the tea pigments and color attributes during the fermentation of YCBT. In addition, a correlation analysis was carried out to clarify the significant correlations between the quality components and the liquor color/appearance attributes. Furthermore, a stepwise regression analysis was used to establish a quantitative prediction model for the tea pigments and identify the key color attribute index. This result could reduce the current misconception that “tea can only be made using empirical observation” during black tea processing. These objective quantitative indicators can quickly be detected by machines, improving the accuracy of the determination of the fermentation degree and leading to more accurate processing.

## 2. Materials and Methods

### 2.1. Materials and Reagents 

Fresh shoots of large-leaf tea cultivated in Mengku (*Camellia sinensis* L.) were plucked in April 2019 from the Yunnan Mengku production area. The moisture content of the fresh tea leaves was ~73%. Each shoot consisted of two leaves and one bud. 

Ethyl acetate, ethyl alcohol, sodium bicarbonate, oxalic acid, and *n*-butyl alcohol (all analytical-grade purity) were purchased from the Shanghai Macklin Biological Technology Company (Shanghai, China).

### 2.2. Tea Processing 

Tea processing was carried out over four key steps, and the leaves were often turned by hand to maintain uniformity. In Step 1 (withering), the fresh leaves (35 kg) were withered (4–5 cm thickness) in an artificial climatic incubator at 28 °C, 65–70% relative humidity, and 3000 lux illumination intensity until the moisture content reached 62–64%. In Step 2 (rolling), the withered leaves were subjected to rolling for 75 min, initially without pressure for 25 min, and then with a light pressure (dropping the rolling machine to make light contact with the leaves) for 20 min, followed by heavy pressure (dropping the rolling machine to press the leaves until no further downward pressure could be exerted) for 10 min, thereafter a light pressure again for 15 min, and finally without pressure for 5 min. Subsequently, in Step 3 (fermenting), the rolled leaves were divided into four equal portions (i.e., A: CK, indoor natural fermentation; B: 25 °C; C: 30 °C; or D: 35 °C) and treated at the specified fermentation temperature in the artificial climate box, where the relative humidity was maintained at 95%. In treatment A, the leaves were fermented using indoor natural fermentation to simulate the common fermentation methods. The fermented leaves were collected after 2.5, 3.5, 4.5, and 5.5 h for each set of fermentation experiments. Finally, in Step 4 (drying), the fermented leaves were dried at 110 °C for 15–20 min until the moisture content reached 20–25%. At this point, the leaves were spread out for a further 30 min at 25 °C, and the samples were dried at 90 °C for 30 min to obtain tea samples containing ~5% moisture. The tea processing was replicated three times. 

### 2.3. Analyses of the Objective Indicators

#### 2.3.1. Detection of the Appearance and Color Attributes of the Tea Samples

During sampling, the appearance color of each sample was triangulated using a portable colorimeter (CM-600d, Konica Minolta Investment Co. Ltd., Shanghai, China) to obtain the color difference attributes that could be used to reflect the degree of fermentation [24]. For this purpose, the *Lab* color difference system was employed, where the *L* value represents the brightness, the *a* value represents the degree of red or green (+ for red, − for green), and the *b* value represents the degree of yellow or blue (+ for yellow, − for blue). In addition, *∆L, ∆a*, and *∆b* represent the chromatic aberrations, while the *∆E* value represents the total chromatic aberration, as per the following formulae [Equations (1)–(4)]:

*∆L* = *L_M_* − *L*_0_; (1)

*∆a* = *a_M_* − *a*_0_; (2)

*∆b* = *b_M_* − *b*_0_; (3)

*∆E* = (*∆L*^2^ + *∆a*^2^ + *∆b*^2^)^1/2^, (4)

in which *L_M_* is the *L* value of the fermented sample and *L*_0_ is the *L* value of the rolled sample; *a_M_* is the *a* value of the fermented sample, *a*_0_ is the *a* value of the rolled sample; and *b_M_* is the *b* value of the fermented sample, while *b*_0_ is the *b* value of the rolled sample.

In addition, the *C* value is the color saturation value, which reflects the gaily colored degree. More specifically, a larger value of *C* correlates with a brighter color. Furthermore, the *H* value represents the hue angle, where 0° is pure red, 45° is orange, 90° is yellow, and 180° is pure green. The instant color differences were measured using the colorimeter at each sampling time, and each measurement was replicated five times.

#### 2.3.2. Detection of the Liquor Color Attributes of the Tea Samples

A sample (3 g) of the unground, dried product was placed in an evaluation cup, and boiling water (150 mL) was added. After allowed to brew for 5 min, the sample was filtered using a tea sieve and filter paper to provide a mother solution tea liquor for testing. More specifically, the obtained liquor was placed in a colorimetric cell of a Konica Minolta tabletop spectrophotometer (CM-5 type, Shanghai, China) to measure its *LL* (liquor translucent degree), *La* (liquor red-green degree), and *Lb* (liquor yellow-blue degree) values [25]. The determination of each value was repeated three times for each sample.

### 2.4. Determination of the TFs, TRs, TBs, Liquor Brightness (BT), and Total Liquor Color (TLC) 

The contents of the various tea pigments were quantified by means of a systematic analysis using a range of organic solvents for extraction (i.e., ethyl acetate, ethyl alcohol, and *n*-butyl alcohol) [16].

Firstly, the unground tea sample (3 g) was placed into a 250 mL conical flask with boiling water (125 mL) and allowed to leach in a boiling water bath for 10 min (shaking 1–2 times). Subsequently, the tea beverage was immediately filtered and quickly cooled under a flow of tap water. To obtain solution O, two milliliters of the cooled tea beverage was mixed with distilled water (8 mL).

Secondly, 25 mL of the tea beverage was placed in a 60 mL separation funnel and ethyl acetate (25 mL) was added. After shaking for 5 min and allowing to separate, the aqueous (lower) layer and the ethyl acetate (upper) layer were collected. Four milliliters and two milliliters of the ethyl acetate (upper) layer were then mixed with 95% ethyl alcohol to make solutions A1 and A2 (25 mL total volume), respectively. Additionally, 15 mL of the ethyl acetate layer was mixed with a 2.5% sodium bicarbonate solution (15 mL), shaken for 30 s, and then the sodium bicarbonate layer was immediately discarded, and an aliquot (4 mL) of the ethyl acetate layer was mixed with 95% ethyl alcohol to make solution B (25 mL total volume). 

Thirdly, two aliquots of the aqueous (lower) layer (both 2 mL) were separated, one was mixed with distilled water (8 mL) and 95% ethyl alcohol to make solution C1 (25 mL final volume), and the other was mixed with a saturated oxalic acid solution (2 mL), distilled water (6 mL), and 95% ethyl alcohol to make solution C2 (25 mL total volume). Moreover, 15 mL of the tea beverage was placed in a 60 mL separation funnel with *n*-butyl alcohol (15 mL), shaken for 3 min, and allowed to separate. An aliquot (2 mL) of the aqueous (lower) layer was mixed with a saturated oxalic acid solution (2 mL), distilled water (6 mL), and 95% ethyl alcohol to make solution D (25 mL).

Using the 95% ethyl alcohol solution as a reference, the absorbances of the above solutions were recorded at 380 and 460 nm [26]. In addition, the optical density (OD) values of the four solutions were measured at 380 nm and were recorded as EA, EB, EC, ED, and EO, which allowed the TFs, TRs, TBs, 10TFRB, BT, and TLC values to be calculated as follows [Equations (5)–(10)]:

TFs (%) = EB_380_ × 2.25 × 3/(M × DM); (5)

TRs (%) = (125 × 0.02 × 6.25[2EC2_380_ + EA1_380_ − EB_380_])/(0.733 × 3 × DM/100); (6)

TBs (%) = 2 × ED_380_ × 7.06 × 3/(M × DM); (7)

10TFRB = (10TFs + TRs)/TBs;(8)

BT (%) = (100 × EB_460_)/(EA1_460_ + EC1_460_); (9)

TLC (%) = EO_460_ × 5 × 3/(M × DM), (10)

where EO is the absorption value of solution O, EA is the absorption value of solution A, EB is the absorption value of solution B, EC is the absorption value of solution C, ED is the absorption value of solution D, and DM is the dry matter mass ratio.

### 2.5. Statistical Analysis

All tests and measurements were repeated in triplicate. The data are expressed as the average of three replicates and are reported as the mean ± standard deviation. SPSS 22.0 (SPSS, Chicago, IL, USA) and Microsoft Excel 2010 software packages were used to analyze the significant differences among the different treatments, and a regression analysis was conducted using these four factors as the target values [16].

## 3. Results and Discussion

### 3.1. Effect of the Fermentation Temperature and Time on the Appearance Color Attributes

**Figure 1:**
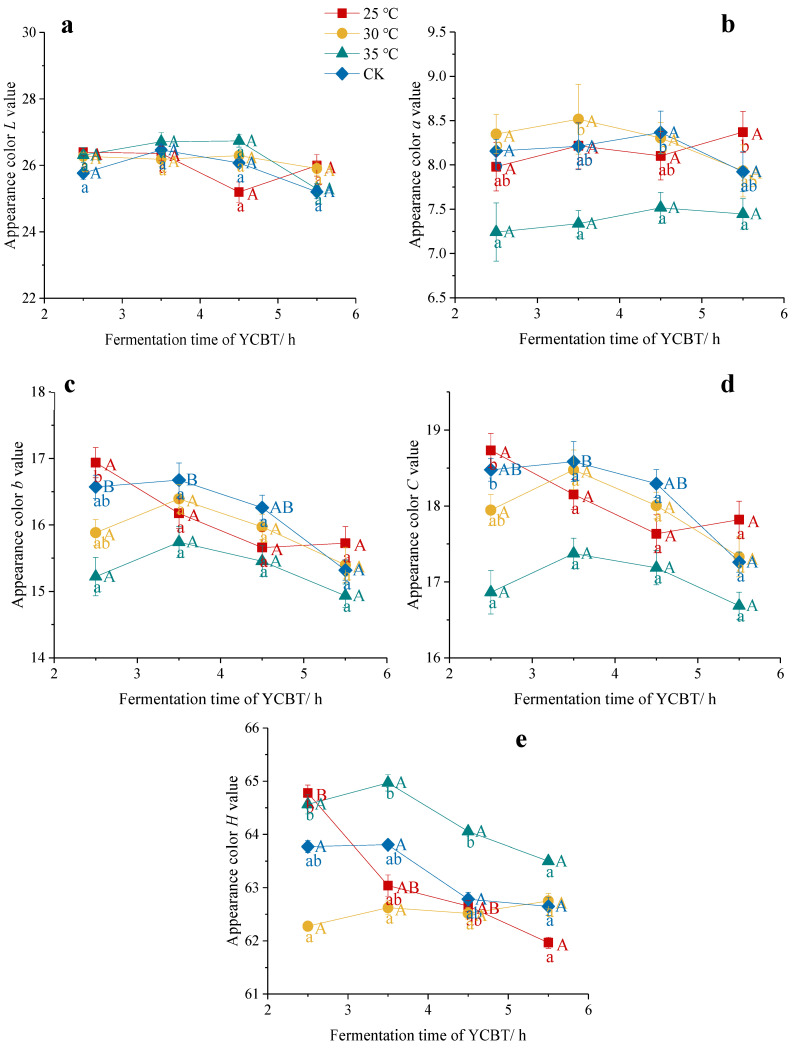
Effects of the fermentation temperature and time on the appearance attributes of Yunnan Congou black tea (YCBT) fermentation: (**a**) *L* value, (**b**) *a* value, (**c**) *b* value, (**d**) *C* value, and (**e**) *H* value. Note: Mean values with different lowercase letters indicate significant differences between different fermentation temperatures at the same fermentation time (*p* < 0.05); Mean values with different capital letters indicate significant differences between different fermentation times at the same fermentation temperature (*p* < 0.05).

The effects of the fermentation temperature and time on the appearance color indices were found to differ significantly among the attributes. As shown in Figure 1, during fermentation, the *L* value of the fermented tea leaves maintained a relatively stable value, and little differences were observed among the four fermentation temperatures. The *b*, and *C* values tended to increase initially and then fluctuate smoothly throughout the remainder in the CK treatment of the fermentation process, which is consistent with the change in color of the fermentation leaves from green to yellow. Upon comparison of the results obtained at the different temperatures, it was apparent that the *L* and *H* values of the fermentation leaves were higher at 35 °C than in other treatments at 3.5 h and 4.5 h; however, there was no significant difference in the *L* value. The *a* value at 30 °C was significantly higher than that at 35 °C (*p* < 0.05) at the same time (within 4.5 h). That is, a moderate increase in temperature favored the generation of the dominant red substances, such as the TFs and the TRs, which could be further polymerized into TBs at higher temperatures, leading to a lower redness value [27]. More specifically, according to each appearance color attribute value of the different fermentation times, the *b* and *C* values at 5.5 h were significantly lower (*p* < 0.05) than at 3.5 h in the CK treatment. These observations, therefore, indicated that the extended times and high temperatures during fermentation led to a reduction in the brightness, and yellowness of the fermentation leaves. However, it should be noted that these results differ from those of previous studies [16], which suggested that the different tea pigment compositions of Yunnan large-leaf tea resulted in different changes in the appearance color attributes during fermentation [28].

**Figure 2:**
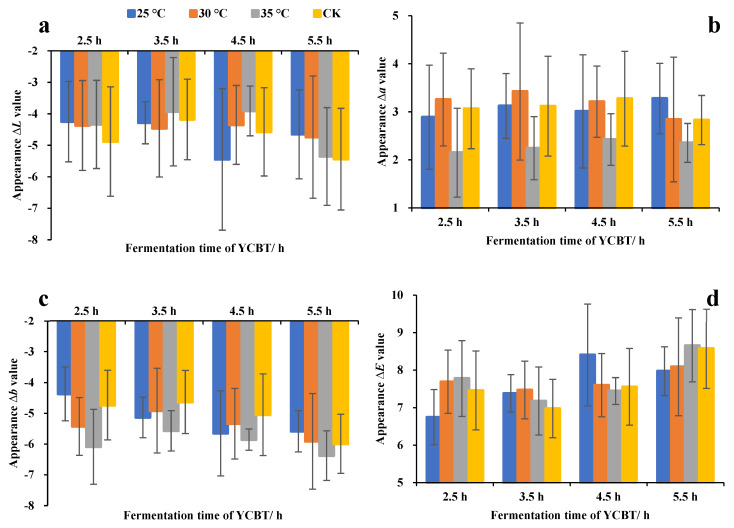
Effect of the fermentation temperature and time on the appearance color of Yunnan Congou black tea (YCBT) fermentation: (**a**) difference in the *L* value between rolled and fermenting leaves, (**b**) difference in the *a* value between rolled and fermenting leaves, (**c**) difference in the *b* value between rolled and fermenting leaves, and (**d**) difference in the total chromatic aberration value between rolled and fermenting leaves.

The variations in the relative color values (i.e., *∆L, ∆a*, and *∆b*) of the fermented leaves can be seen in Figure 2, and there were no significant difference results for time and temperature, which showed that the fermentation process was well-distributed and relatively uniform. Compared with the rolled leaves, the *∆L* value was always negative during the fermentation process (Figure 2a) and, combined with the *L* value, it indicated the fermentation was adverse to maintaining the product brightness [29]. In addition, the *∆a* value was positive during fermentation and, combined with the *a* value, its meant that high temperatures were not suitable for fermenting leaves turning red (Figure 2b). The fermentation process generally had a negative impact on the *∆b* value (Figure 2c), proving that fermented leaves had difficulty retaining the yellow value attribute in fermentation, especially at high temperatures, which is consistent with the existing research [16]. The value of *∆E* was positive during the fermentation process (Figure 2d) and, combined with the appearance color values, it was considered that the color attributes of the leaves during fermentation changed greatly compared with the rolling process. It was revealed that the positive or negative effects of the leaves color attributes could be regulated by temperature and time in the fermentation process.

### 3.2. Effects of the Fermentation Temperature on the Liquor Color Attributes

**Figure 3:**
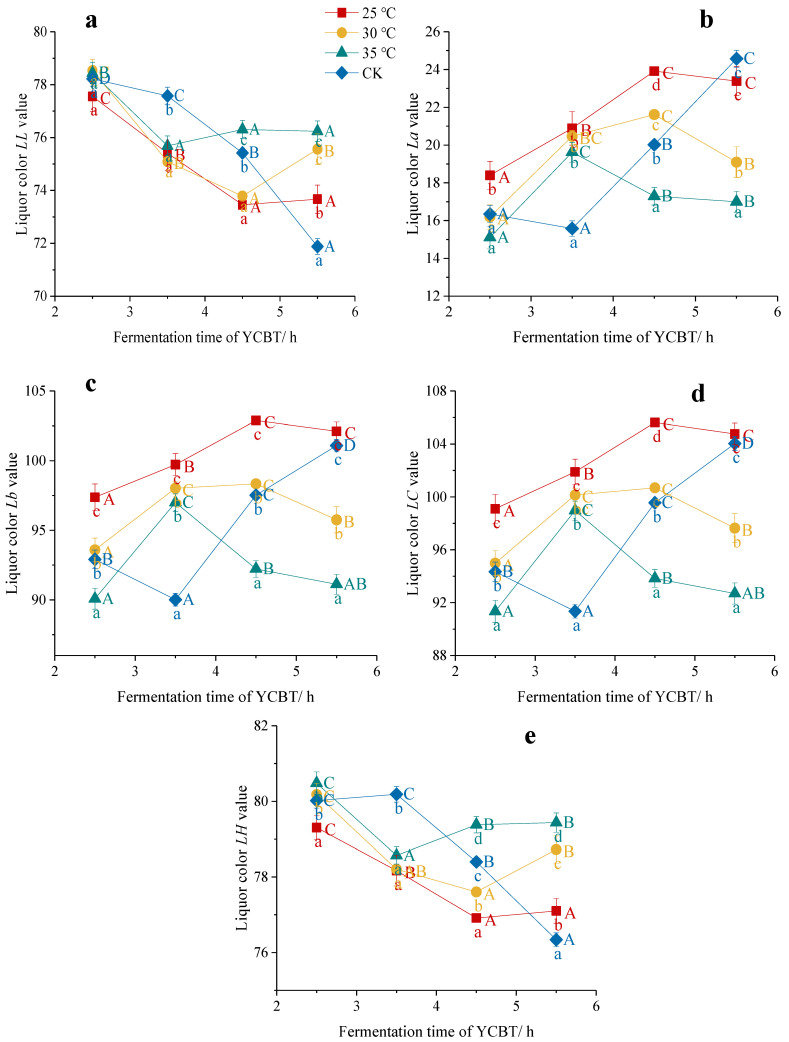
Effect of the fermentation temperature and time on the liquor color of Yunnan Congou black tea (YCBT) fermentation: (**a**) *LL* value, (**b**) *La* value, (**c**) *Lb* value, (**d**) *LC* value, and (**e**) *LH* value. Note: Mean values with different lowercase letters indicate significant differences between different fermentation temperatures at the same fermentation time (*p* < 0.05); Mean values with different capital letters indicate significant differences between different fermentation times at the same fermentation temperature (*p* < 0.05).

As shown in Figure 3, during the fermentation process, the *LL* (Figure 3a) and *LH* (Figure 3e) values of the black tea liquor gradually decreased, and the values increased after reaching the lowest value after 3.5 h at 35 °C, while being delayed to 4.5 h in the lower temperature treatments (30 °C, *p* < 0.05). In addition, the *La*, *Lb*, and *LC* values initially increased until 4.5 h, and then decreased in the 30 °C treatment, while they increased until 3.5 h in the higher treatment (35 °C, *p* < 0.05). This is consistent with the phenomenon that the liquor gradually becomes red in color due to the gradual accumulation of TRs [30]. The CK treatment initially decreased slightly and then increased linearly, which indicated that biochemical reactions lagged under CK conditions due to the lower temperatures in the early stages of the CK experiment [16]. Overall, the values of *La*, *Lb*, and *LC* (*p* < 0.05) appeared to be optimized at a low fermentation temperature (25 °C, 30 °C) before 4.5 h, and these values decreased after increasing the temperature. Thus, high-temperature fermentation led to a reduced redness, yellowness, and saturation of the tea liquor, while low-temperature fermentation was beneficial to the enrichment of yellow and red substances, such as the TFs and TRs, to produce a bright red liquor color.

### 3.3. Effect of the Fermentation Temperature on the TFs, TRs, TBs, and Other Mass Fractions

**Figure 4:**
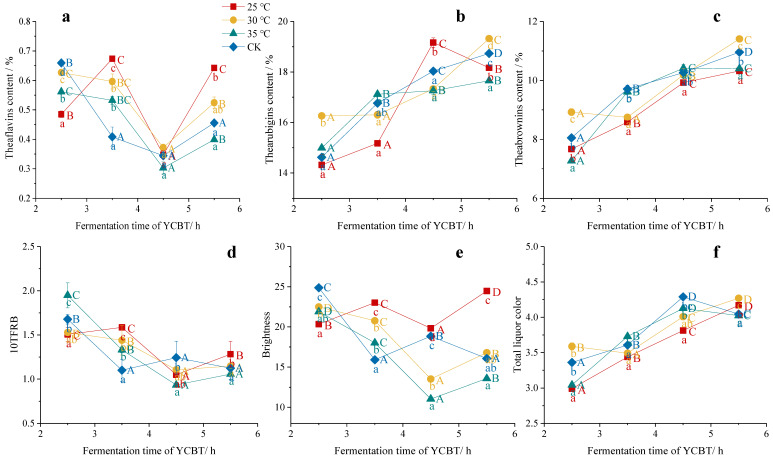
Effect of the fermentation temperature and time on the tea pigment contents of Yunnan Congou black tea (YCBT) fermentation: (**a**) TFs content, (**b**) TRs content, (**c**) TBs content, (**d**) 10TFRB value, (**e**) BT value, and (**f**) TLC value. Note: Mean values with different lowercase letters indicate significant differences between different fermentation temperatures at the same fermentation time (*p* < 0.05); Mean values with different capital letters indicate significant differences between different fermentation times at the same fermentation temperature (*p* < 0.05).

The TFs have the most significant effect on the brightness of tea liquor [31]. As shown in Figure 4a, during the fermentation process, the maximum accumulation of TFs was observed after 3.5 h at 25 °C, beyond which point the TFs content decreased due to their conversion into other compounds [11]. At a higher fermentation temperature of 35 °C, the TFs content showed a significant decline trend during the late stage of fermentation (after 3.5 h, *p* < 0.05) [32]. Therefore, these results suggest that a low fermentation temperature (i.e., 25 °C) is the most conducive to the continuous formation of TFs. 

Similarly, the variation in the TRs content at different fermentation temperatures and times was examined, as presented in Figure 4b. More specifically, at 25 °C, the greatest degree of TRs formation and accumulation was observed at 4.5 h; at 35 °C, the TRs content did not increase significantly after 3.5 h of fermentation, while the TRs value kept increasing in the whole fermentation process at 30 °C and with the CK treatments. In terms of the effects of these differences on the final tea product, it has been reported that the TRs content is responsible for the red color of black tea liquor and the concentration and strength of the black tea taste [33]. Our results showed a clear increase in the TRs content in the initial stages of fermentation upon increasing the temperature and duration, which is in agreement with the results of a previous study by Muthumani and Kumar [34].

The other key compounds in black tea are the TBs, which tend to give the liquor a dark brown color and a flat taste, which ultimately has an adverse effect on the quality of black tea. As presented in Figure 4c, the TBs exhibited an increasing trend during the whole fermentation process, which was consistent with the findings of Hua [16]. More specifically, it was found that the TBs content initially increased gradually upon fermentation at 25 and 30 °C, but increased more rapidly between 3.5 and 4.5 h, which corresponded to the changes in the TFs and TRs contents. This is because TFs and TRs were formed continuously at low temperatures, which led to a slow increase in the TBs content in the early stages of a low-temperature fermentation. In contrast, during fermentation at 35 °C, the TBs content increased rapidly in the initial 4.5 h, and then increased slightly in the later stage. Therefore, high fermentation temperatures favored high TBs production due to the polymerization of TFs and TRs.

**Figure 5:**
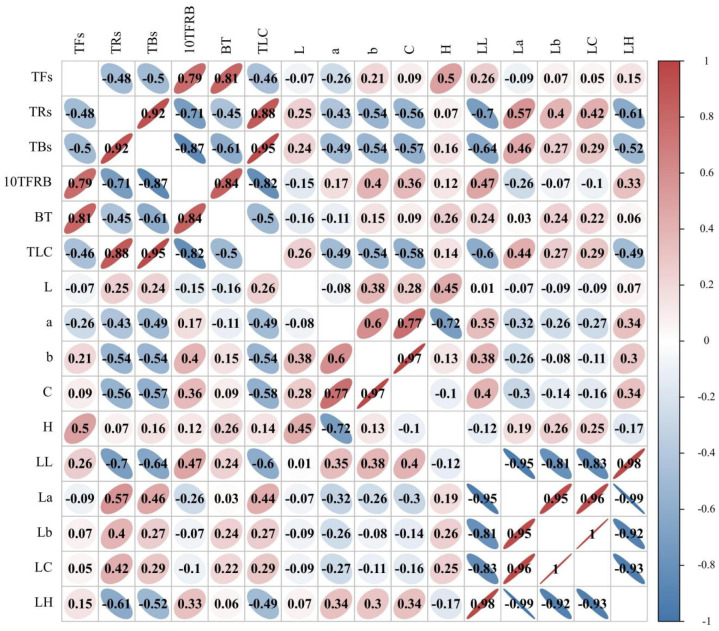
Correlation analysis between the color attributes and the tea pigment contents of fermented leaves.

Generally, TFs, TRs, and TBs have a competitive relationship [35]. When considering the same substrate, a low fermentation temperature (i.e., 25 or 30 °C) was found to be beneficial to the continuous formation of TFs and TRs, and their further conversion to TBs via polymerization was limited. To thoroughly reflect the influence of the fermentation temperature and time on the transformation of these three tea pigments, a comprehensive evaluation index of 10TFRB tea pigments was introduced [36], wherein 10TFRB represents the ratio of the sum of 10 times the TFs and TRs to the TBs. BT and TLC were also used as comprehensive indicators of tea pigments, but as shown in Figure 5, the two indicators had no significant correlation with TFs and TRs. Although 10TFRB had a higher value in the early stage of fermentation, a short fermentation time was not conducive to improving the taste quality of black tea, which was found to be optimal after 3.5 h. As shown in Figure 4d, the 10TFRB value initially increased then decreased at low fermentation temperatures (i.e., 25 or 30 °C), and decreased steadily at 30 °C and with the CK treatment during the fermentation process. This result indicates that the formation of TFs and TRs is continuous under low-temperature fermentation [37], while the TBs content increases rapidly at higher temperatures [38]. Therefore, we deduced that a temperature of 30 °C and a fermentation time of 3.5 h were the most suitable conditions for the formation of desirable YCBT quality components (i.e., the TFs and TRs).

### 3.4. Correlation Analysis among the Appearance Color Attributes, Liquor Color Attributes, and Tea Pigments

TFs are important components in developing the freshness and strength of the black tea taste. As shown in Figure 5, the TFs were significantly positively correlated with the BT and *H* values, which is consistent with previous studies [39]. However, the TFs showed no significant correlations with any other pigment values, although they were positively correlated with the *b* and *LL* values, and negatively correlated with the *a* value. These results suggest that a low-temperature fermentation, which promotes TFs formation, was more beneficial to the formation of the orange appearance attribute and the bright liquor color attribute. 

The TRs are the main substances responsible for the color of black tea liquor, and they are also known to affect taste, strength, and convergence [40]. The results presented in Figure 5 therefore show that the TRs were significantly positively correlated with the TLC and *La* values, but negatively correlated with the *b*, *C*, *LL*, and *LH* values. Therefore, the optimal liquor redness can be achieved under low-temperature fermentation conditions where greater quantities of TRs are formed.

The TBs are dark brown in color and are important components in determining the dark and nonconvergence properties of tea liquor [39]. As shown in Figure 5, the TBs content was significantly positively correlated with the TLC value, but negatively correlated with the BT, *b*, *C*, *LL*, and *LH* values. More specifically, high TBs contents would cause the tea liquor to have low brightness and transparency properties, as well as a poor yellowness and glossiness degree of appearance. In addition, the data presented in Figure 5 show that a low-temperature fermentation prevented the decomposition of TRs into TBs, which could lead to a brighter tea liquor. However, the TLC value was only significantly positively correlated with the TRs and TBs, so the traditional total liquor color value was more inclined to the darker values, which did not fully reflect the effects of the three kinds of tea pigments.

Moreover, the 10TFRB value was significantly positively correlated with the BT value and TFs content but was significantly negatively correlated with the TRs and TBs contents in addition to the TLC (*p* < 0.01, R^2^ > 0.7). BT and TLC were also used as comprehensive indicators of tea pigments [41], but as shown in Figure 5, the two indicators had no significant correlation with TFs and TRs. We then speculated that these indexes may not be applicable to YCBT with special tea pigment contents. Thus, the 10TFRB value could effectively represent the contribution of TFs, TRs, and TBs in YCBT, which were consistent with the *LL* value of the liquor color, and maintained its highest values at fermentation temperatures of 25 and 30 °C.

### 3.5. Prediction Model for the TFs, TRs, and TBs Contents, and the 10TFRB Value

The TFs, TRs, and TBs are the key quality and functional components of YCBT, and they are important indicators for judging the moderate fermentation process. However, the existing methods for detecting tea pigments are complex and tend to exhibit poor replicability and low accuracy. As shown in Figure 5, the appearance color attributes and liquor color attributes are significantly correlated with the TFs, TRs, and TBs contents and the 10TFRB value, therefore, a regression analysis was conducted using these four factors as the target values (Table 1). 

When the TFs content was used as the target value, the ratio of error for the dependent variable was too large, which led to the model not reaching significance (*p* = 0.356). A similar situation was also observed for the 10TFRB value (*p* = 0.203), indicating that it was not feasible to establish a linear regression model for predicting the TFs content or the 10TFRB value based on the above attributes as independent variables. In contrast, as shown in Table 1, when the TRs and TBs contents were used as the target value, the models reached significance (*p* < 0.05). Furthermore, a stepwise regression analysis showed that the liquor color *LL* had a significant influence on the TRs content, and the liquor colors *LL* and *LH* had a significant influence on the TBs content (Table 2). In this case, the multiple decision coefficients *R^2^* of 0.670 and 0.669 were obtained. The two multiple linear regression prediction models for TRs and TBs were obtained as follows (Equations (11) and (12)):

TRs = 60.014 − 0.568 V*_LL_*; (11)

TBs = −24.656 − 1.695 V*_LL_* + 2.067V*_LH_*, (12)

where V*_LL_* is the *LL* value of the liquor color brightness, and V*_LH_* is the *LH* value of the liquor color hue angle.

## 4. Conclusions

We clarified the previously unprecedented evolution law of objective quantitative indexes of the appearance and liquor color attributes of Yunnan Congou black tea (YCBT) during the fermentation process. We found that both the fermentation temperature and time had significant effects on the different color indexes and tea pigment components. Overall, a low-temperature fermentation was beneficial to maintaining an orange appearance and bright red liquor of YCBT, and a higher-temperature fermentation was beneficial to increasing the bright appearance and salmon pink liquor color. A period of 3.5–4.5 h was the suitable fermentation time to obtain the bright red appearance and liquor color of YCBT. Furthermore, the developed 10TFRB index can be used for comprehensive evaluations of tea pigments. A correlation analysis showed that the theaflavins (TFs) had a significantly positive correlation with the appearance hue *H* value, while the thearubigins (TRs) had a significantly negative correlation with the liquor translucent degree *LL* value and appearance *b* and *C* values, and a significantly positive correlation with the liquor red degree *La* value, and the theabrownins (TBs) had the same correlation as that of the TRs. Moreover, multiple linear regression models of the TFs and TBs were established based on objective quantitative indicators (i.e., the color attributes), which showed that the liquor *LL* value was the most critical factor and could therefore be used as a key indicator to judge the moderate fermentation process and measure the TRs and TBs contents. Our follow-up study will focus on a further refinement of the evolution association between the polyester catechin, TFs, and TRs components present in YCBT during the fermentation process, and we will also explore how fermentation temperature and time affect their formation.

## Figures and Tables

**Table 1:** Regression analysis of the TFs, TRs, TBs, and 10TFRB values as target values.

Target Value	Source	DF	Sum of Squares	Mean Squares	*F* Value	*p*
TFs	Regression	0.139	8	0.017	1.342	0.356
	Residual	0.091	7	0.013		
	Corresponding total amount	0.230	15			
TRs	Regression	31.659	8	3.957	4.803	0.026
	Residual	5.767	7	0.824		
	Corresponding total amount	37.426	15			
TBs	Regression	18.230	8	2.279	4.784	0.027
	Residual	3.335	7	0.476		
	Corresponding total amount	21.564	15			
10TFRB	Regression	0.657	8	0.082	1.919	0.203
	Residual	0.300	7	0.043		
	Corresponding total amount	0.957	15			

**Table 2:** Significances and parameter estimations of stepwise regression analysis using the TRs and TBs values as target value.

Target Value	Variable	DF	Parameter Estimate	Standard Error	*t* Value	*p*
TRs	Constant variable	1	60.014	11.640	5.156	<0.0001
	LL	1	−0.568	0.154	−3.701	0.002
TBs	Constant variable	1	−24.656	23.298	−1.058	0.309
	LL	1	−1.695	0.457	−3.712	0.003
	LH	1	2.067	0.709	2.917	0.012

## Data Availability

Data is contained within the article.
